# Expected value of first Zagreb connection index in random cyclooctatetraene chain, random polyphenyls chain, and random chain network

**DOI:** 10.3389/fchem.2022.1067874

**Published:** 2023-01-04

**Authors:** Zahid Raza, Shehnaz Akhter, Yilun Shang

**Affiliations:** ^1^ Department of Mathematics, College of Sciences, University of Sharjah, Sharjah, United Arab Emirates; ^2^ School of Natural Sciences, National University of Sciences and Technology, Islamabad, Pakistan; ^3^ Department of Computer and Information Sciences, Northumbria University, Newcastle upon Tyne, United Kingdom

**Keywords:** average value, expected value, random cyclooctatetraene chain, random polyphenyls chain, zagreb connection indices

## Abstract

The Zagreb connection indices are the known topological descriptors of the graphs that are constructed from the connection cardinality (degree of given nodes lying at a distance 2) presented in 1972 to determine the total electron energy of the alternate hydrocarbons. For a long time, these connection indices did not receive much research attention. Ali and Trinajstić [Mol. Inform. 37, Art. No. 1800008, 2018] examined the Zagreb connection indices and found that they compared to basic Zagreb indices and that they provide a finer value for the correlation coefficient for the 13 physico-chemical characteristics of the octane isomers. This article acquires the formulae of expected values of the first Zagreb connection index of a random cyclooctatetraene chain, a random polyphenyls chain, and a random chain network with *l* number of octagons, hexagons, and pentagons, respectively. The article presents extreme and average values of all the above random chains concerning a set of special chains, including the meta-chain, the ortho-chain, and the para-chain.

## 1 Introduction

Graph theory is vital to various disciplines, including the chemical and biological sciences. One of the objectives of chemical graph theory is its primary and significant role in studying physico-chemical reactions and biological activities and pointing out the structural properties of molecular graphs, *etc.,* Topological descriptors have played a significant role in achieving the desired properties of molecular graphs. Topological descriptors are molecular structural invariants that theoretically and mathematically explain the connectivity characteristics of nano-materials and chemical compounds. Therefore, topological indices produce sharper approaches to measuring their behavior and characteristics.

For 20 years, hydrocarbons and their derivatives have received attention from researchers because these compounds only have two members, carbon and hydrogen. We can acquire various types of hydrocarbon derivatives by replacing their molecular hydrogen atoms with different types of other atomic groups. A large number of valuable hydrocarbons are available in plants and some valuable characteristics of hydrocarbons are important to chemical raw materials and fuel.

Throughout this article, the vertex and edge sets of a graph H are represented as V(H) and E(H) , respectively. We denote the degree of a vertex v∈V(H) by $d_{H}\left(v\right)$ , which is defined as the cardinality of edges joined with v . Let the order and size of H be *n* and *m*, respectively. The *l*-degree of a given vertex v∈V(H) , presented by $d_{l}\left(v\right)$ , is the cardinality of set of vertices of V(H) whose distance from v is *l*, where $d_{1}\left(v\right)=d_{H}\left(v\right)$ and $d_{2}\left(v\right)=\tau_{v}$ [this is known as the connection number of v (Todeschini and Consonni, 2000)].

Suppose that Z is a collection of all connected simple graphs. There is a function $P:Z\to R^{+}$ that describes a topological invariant if for any two isomorphic members $M_{1}$ and $M_{2}$ of Z , we have $P\left(M_{1}\right)=P\left(M_{2}\right)$ . Thousands of degree and distance-related topological invariants have been proposed, but some are better known because of their high predictive power for many characteristics like density, boiling point, molecular weight, refractive index, *etc.,* Topological invariants have so many implementations in numerous areas of sciences such as drug discovery, physico-chemical research, toxicology, biology, and chemistry. To date, topological indices are the most notable field of graphical research. For more discussion on numerous invariants, we refer readers to studies by (Gutman, 2013; Akhter et al., 2016; Akhter and Imran, 2016; Akhter et al., 2017; Akhter et al., 2018; Akhter et al., 2020).

The Zagreb indices are the most notable invariants, and they have many valuable applications in chemistry. In 1972 Gutman and Trinajstić (Gutman and Trinajstić, 1972) established the first vertex degree dependent Zagreb index of a graph H . Two renowned Zagreb indices of a graph H can be described in the following manner:

$$\begin{array}{l} M_{1}\left(H\right)=\underset{x\in V\left(H\right)}{\sum }d_{H}^{2}\left(x\right),\\ M_{2}\left(H\right)=\underset{xy\in E\left(H\right)}{\sum }d_{H}\left(x\right)d_{H}\left(y\right). \end{array}$$

Motivated by how influential they have become and the many important applications of primary Zagreb indices, Naji et al. (Soner and Naji, 2016; Gutman et al., 2017) presented the concept of Zagreb connection indices (leap Zagreb indices), constructed from the second degrees of the vertices of a graph H . The first, second, and modified Zagreb connection indices of H can be defined as:

$$\begin{array}{l} ZC_{1}\left(H\right)=\underset{y\in V\left(H\right)}{\sum }\tau_{y}^{2},\\ ZC_{2}\left(H\right)=\underset{\mathrm{xy}\in E\left(H\right)}{\sum }\tau_{x}\tau_{y},\\ ZC_{1}^{*}\left(H\right)=\underset{x\in V\left(H\right)}{\sum }d_{H}\left(x\right)\tau_{x}. \end{array}$$

The chemical applications of *ZC*_1_ were presented in (8), indicating that the given index has a wide co-relation with the physical characteristics of chemical compounds, for instance, boiling point, enthalpy of evaporation, entropy, acentric factor, and standard enthalpy of vaporization. Let *f*_*l*_ present the cardinality of the subset of vertices of H with connection number *l*. The next formula for the first Zagreb connection index is equal to the above definition.

$$ZC_{1}\left(H\right)=\underset{0\leq l\leq n-2}{\sum }f_{l}\left(G\right)l^{2}.$$ (1.1)

Naji and Soner (2018), (Gutman et al., 2017) determined the leap Zagreb descriptors of some graph operations and families. Leap Zagreb indices are presented in a recently published survey (Gutman et al., 2020). In (39), the authors establish sharp bounds for the leap Zagreb indices of trees and unicyclic graphs and also determined the corresponding extremal graphs. For more studies on Zagreb connection indices, we refer the readers to (Ducoffe et al., 2018a; Ali and Trinajstić, 2018; Shao et al., 2018a; Basavanagoud and Chitra, 2018; Ducoffe et al., 2018b; Khalid et al., 2018; Manzoor et al., 2018; Du et al., 2019; Fatima et al., 2019; Tang et al., 2019; Ye et al., 2019; Raza, 2020a; Bao et al., 2020; Raza, 2020b; Cao et al., 2020; Naji et al., 2020; Raza, 2022).

Huang et al. (2014) determined the expected values for Kirchhoff indices of random polyphenyl and spiro chains. Ma et al. (2017), Yang and Zhang. (2012), and Qi et al. (2022) independently acquired the expected value of Wiener indices of random polyphenyl chain and random spiro chain. Zhang et al. (2020) have provided expected values of the Schultz, Gutman, multiplicative degree-Kirchhoff, and additive degree-Kirchhoff indices of random polyphenylene chains. Raza and Imran. (2021) obtained expected values of modified second Zagreb, symmetric difference, inverse symmetric, and augmented Zagreb indices in random cyclooctane chains. Zhang et al. (2021) established the formulae for expected values of Sombor indices of a general random chain. Recently, many studies have explored the expected values of different topological indices. For further information, we refer readers to the following studies (Raza, 2020b; Fang et al., 2021; Raza, 2021; Jahanbanni, 2022; Raza et al., 2022).

Motivated by the above research, the present study determined the explicit formulae for expected values of the first Zagreb connection index of the random cyclooctatetraene chain, random polyphenyls chain, and random chain network with *l* octagons, hexagons, and pentagons, respectively. Moreover, we examined the average and extreme values of the Zagreb connection index among all the above-mentioned random chains corresponding to their set.

## 2 The first Zagreb connection index of random cyclooctatetraene chain

Cyclooctatetraene, having chemical formula *C*_8_ *H*_8_, is an organic compound whose full name is ‘1, 3, 5, 7 − cyclooctene. Its structure is a cyclic polyolefin-like benzene, but it is not aromatic, see (Willis et al., 1952; Mathews and Lipscomb, 1959; Traetteberg et al., 1970). It has the same chemical characteristics as unsaturated hydrocarbons and is easy to construct explosive organic peroxides, (Milas and NolanPetrus, 1958; Donald and Whitehead, 1969; Garavelli et al., 2002; Schwamm et al., 2019).

**FIGURE 1:**
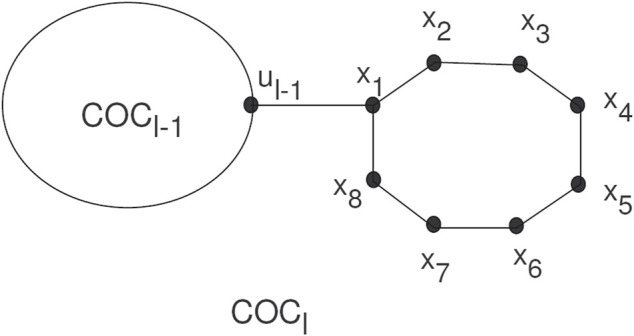
A random cyclooctatetraene chain *COC*_*l*_.

Spiro compounds are valuable types of cycloaltanes in organic chemistry. A spiro union is a join of two rings that have a common atom between both rings, and a join of a direct union among the rings is known as a free spiro union in spiro compounds. In a cyclooctatylene chain, octagons are joined by cut vertices or cut edges. A random cyclooctatetraene chain *COC*_*l*_, has *l* octagons, and can be constructed by a cyclooctatetraene chain *COC*_*l*−1_ with *l*−1 octagons attached to a new octagon *G*_*l*_ by a bridge (see Figure 1).

**FIGURE 2:**
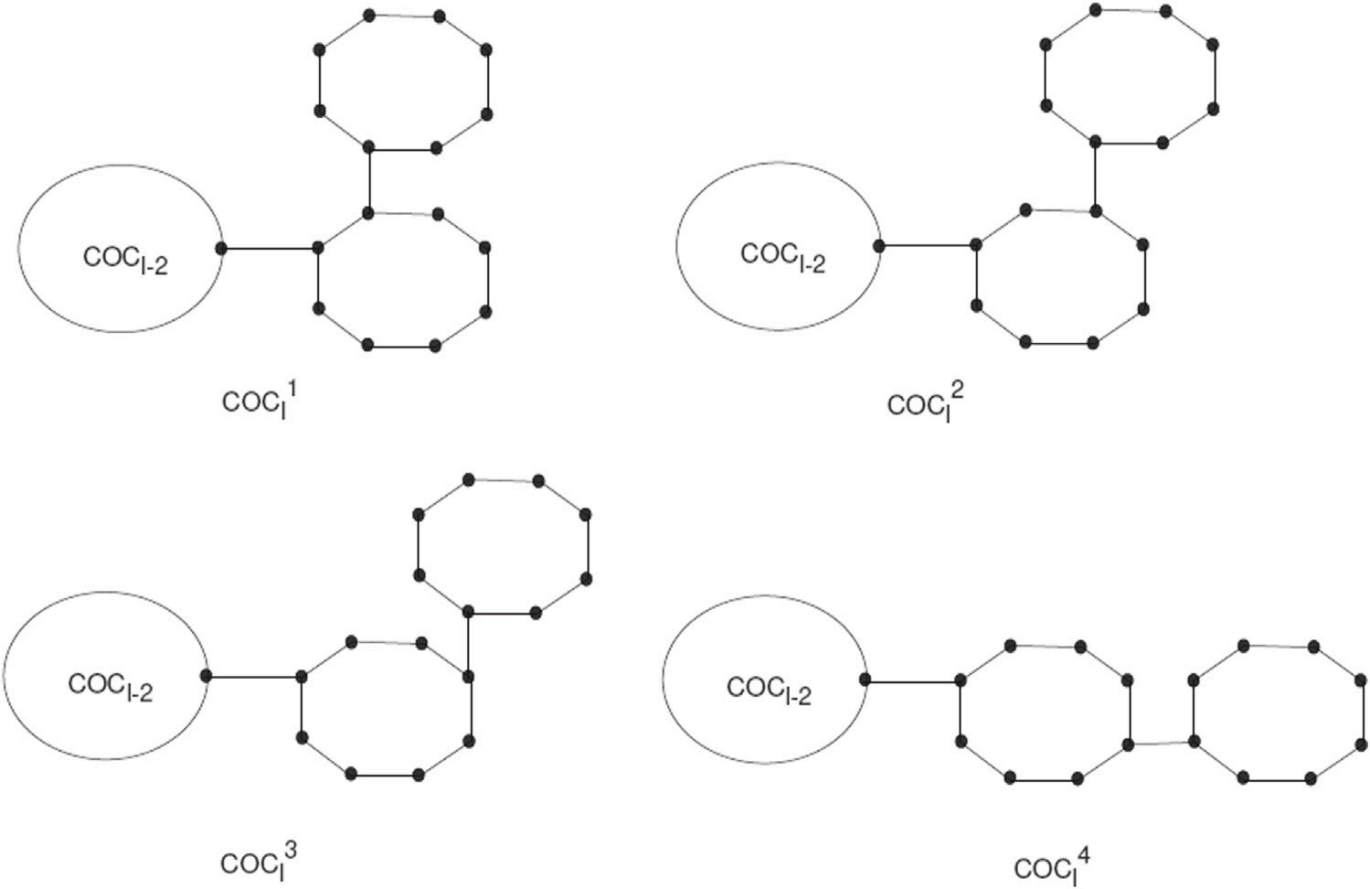
The four types of local arrangements of octagons $COC_{l}^{1}$ , $COC_{l}^{2}$ , $COC_{l}^{3}$ and $COC_{l}^{4}$ .

The *COC*_*l*_ is a cyclooctatetraene chain with *l* ≥ 2 having *G*_1_, *G*_2_, *…* , *G*_*l*_ octagons. The new octagon can be joined by four different schemes, which give the local orderings. We use these as $COC_{l}^{1}$ , $COC_{l}^{2}$ , $COC_{l}^{3}$ , $COC_{l}^{4}$ (see Figure 2).

A random cyclooctatetraene chain *COC*_*l*_(*k*_1_, *k*_2_, *k*_3_) is a cyclooctatetraene chain constructed by step-by-step attachment of new octagons. At every step *p* = 2, 3, *…* , *l* a random choice is constructed from one of the four possible chains:

1 $COC_{p-1}\to COC_{p}^{1}$ with probability *k*_1_,
2 $COC_{p-1}\to COC_{p}^{2}$ with probability *k*_2_,
3 $COC_{p-1}\to COC_{p}^{3}$ with probability *k*_3_,
4 $COC_{p-1}\to COC_{p}^{4}$ with probability *k*_4_ = 1 − *k*_1_−*k*_2_−*k*_3_,

Where all the given probabilities are constant. In this section, we will discuss the expected value for the first Zagreb connection index among random cyclooctatetraene chains with *l* octagons.

Theorem 2.1 *For* *l* ≥ 2*, the expected value for the first Zagreb connection index of random cyclooctatetraene chain* *COC*_*l*_ *is*

$$E\left(ZC_{1}\left(COC_{l}\right)\right)=\left(8k_{1}+2k_{2}+76\right)l-16k_{1}-4k_{2}-44.$$

Proof. **Case-I:** When *l* = 2, we get the result by direct calculations as:

$$E\left(ZC_{1}\left(COC_{l}\right)\right)=10\times {\left(2\right)}^{2}+4\times {\left(3\right)}^{2}+2\times {\left(4\right)}^{2}=108.$$

**Case-II:** When *l* ≥ 3, it is obvious that *f*_2_(*COC*_*l*_), *f*_3_(*COC*_*l*_), *f*_4_(*COC*_*l*_) and *f*_5_(*COC*_*l*_) depends on the four possible cases as following:

1 If $COC_{l-1}\to COC_{l}^{1}$ with probability *k*_1_, we acquire

$$\begin{array}{ll} f_{2}\left(COC_{l}^{1}\right) & =f_{2}\left(COC_{l-1}\right)+4,\\ f_{3}\left(COC_{l}^{1}\right) & =f_{3}\left(COC_{l-1}\right)+2,\\ f_{4}\left(COC_{l}^{1}\right) & =f_{4}\left(COC_{l-1}\right)=2,\\ f_{5}\left(COC_{l}^{1}\right) & =f_{5}\left(COC_{l-1}\right)+2. \end{array}$$

By using the above values in Eq. 1.1 , we get

$$\begin{array}{ll} ZC_{1}\left(COC_{l}^{1}\right) & =ZC_{1}\left(COC_{l-1}\right)+4\times 2^{2}\\ & \;+2\times 3^{2}+2\times 5^{2}\\ & =ZC_{1}\left(COC_{l-1}\right)+84. \end{array}$$

2 If $COC_{l-1}\to COC_{l}^{2}$ with probability *k*_2_, we acquire

$$\begin{array}{ll} f_{2}\left(COC_{l}^{2}\right) & =f_{2}\left(COC_{l-1}\right)+3,\\ f_{3}\left(COC_{l}^{2}\right) & =f_{3}\left(COC_{l-1}\right)+2,\\ f_{4}\left(COC_{l}^{2}\right) & =f_{4}\left(COC_{l-1}\right)+3, \end{array}$$

By using the above values in Eq. 1.1, we get

$$\begin{array}{ll} ZC_{1}\left(COC_{l}^{2}\right) & =ZC_{1}\left(COC_{l-1}\right)+3\times 2^{2}+2\times 3^{2}\\ & \;+3\times 4^{2}\\ & =ZC_{1}\left(COC_{l-1}\right)+78. \end{array}$$

3 If $COC_{l-1}\to COC_{l}^{3}$ with probability *k*_3_, we acquire

$$\begin{array}{ll} f_{2}\left(COC_{l}^{3}\right) & =f_{2}\left(COC_{l-1}\right)+2,\\ f_{3}\left(COC_{l}^{3}\right) & =f_{3}\left(COC_{l-1}\right)+4,\\ f_{4}\left(COC_{l}^{3}\right) & =f_{4}\left(COC_{l-1}\right)+2, \end{array}$$

By using the above values in Eq. 1.1, we get

$$\begin{array}{ll} ZC_{1}\left(COC_{l}^{3}\right) & =ZC_{1}\left(COC_{l-1}\right)+2\times 2^{2}+4\times 3^{2}\\ & \;+2\times 4^{2}\\ & =ZC_{1}\left(COC_{l-1}\right)+76. \end{array}$$

4 If $COC_{l-1}\to COC_{l}^{4}$ with probability 1 − *k*_1_−*k*_2_−*k*_3_, we acquire

$$\begin{array}{ll} f_{2}\left(COC_{l}^{4}\right) & =f_{2}\left(COC_{l-1}\right)+2,\\ f_{3}\left(COC_{l}^{4}\right) & =f_{3}\left(COC_{l-1}\right)+4,\\ f_{4}\left(COC_{l}^{4}\right) & =f_{4}\left(COC_{l-1}\right)+2, \end{array}$$

By using above the values in Eq. 1.1, we get

$$\begin{array}{ll} ZC_{1}\left(COC_{l}^{4}\right) & =ZC_{1}\left(COC_{l-1}\right)+2\times 2^{2}+4\times 3^{2}\\ & \;+2\times 4^{2}\\ & =ZC_{1}\left(COC_{l-1}\right)+76. \end{array}$$

Now

$$\begin{array}{ll} E_{l}^{i}=E\left(ZC_{1}\left(COC_{l}\right)\right) & =k_{1}ZC_{1}\left(COC_{l}^{1}\right)+k_{2}ZC_{1}\left(COC_{l}^{2}\right)+k_{3}ZC_{1}\left(COC_{l}^{3}\right)\\ & \;+\left(1-k_{1}-k_{2}-k_{3}\right)ZC_{1}\left(COC_{l}^{4}\right)\\ & =ZC_{1}\left(COC_{l-1}\right)+8k_{1}+2k_{2}+76. \end{array}$$ (2.1)

Note that $E\left[E_{l}^{i}\right]=E_{l}^{i}$ . By applying the expression operator to Eq. 2.1 and also *l* ≥ 3, we get

$$E_{l}^{i}=E_{l-1}^{i}+8k_{1}+2k_{2}+76.$$ (2.2)

The Eq. 2.2 is a first-order non-homogeneous linear difference result with constant coefficients. We easily see that the general solution of the homogeneous equation of Eq. 2.2 is *E* ^*i*^ = *C*. Suppose *E* ^*i*^′ = *bl* is a particular solution of Eq. 2.2, using *E* ^*i*^′ into Eq. 2.2, we acquire

$$b=8k_{1}+2k_{2}+76.$$

Finally the general solution of Eq. 2.2 is

$$\begin{array}{ll} E_{l}^{i} & =E^{i}+E^{i^{\prime }}\\ & =E\left(ZC_{1}\left(COC_{l}\right)\right)=\left(8k_{1}+2k_{2}+76\right)l+C. \end{array}$$

Applying the initial condition *l* = 3, we get the following

$$C=-16k_{1}-4k_{2}-44.$$

Therefore

$$\begin{array}{ll} E_{l}^{i} & =E\left(ZC_{1}\left(COC_{l}\right)\right)\\ & =\left(8k_{1}+2k_{2}+76\right)l-16k_{1}-4k_{2}-44. \end{array}$$

If *k*_1_ = 1 (respectively, *k*_2_ = 1) and *k*_2_ = *k*_3_ = *k*_4_ = 0 (respectively, *k*_1_ = *k*_3_ = *k*_4_ = 0), then *COC*_*l*_ = *M*_*l*_ (respectively, $COC_{l}=O_{l}^{1}$ ). Similarly, if *k*_3_ = 1 (respectively, *k*_4_ = 1) and *k*_1_ = *k*_2_ = *k*_4_ = 0 (respectively, *k*_1_ = *k*_2_ = *k*_3_ = 0), then $COC_{l}=Q_{l}^{2}$ (respectively *COC*_*l*_ = *L*_*l*_). By Theorem 2.1, we can acquire the first Zagreb connection index of the cyclooctatetraene meta-chain *M*_*l*_, ortho-chains $O_{l}^{1}$ , $O_{l}^{2}$ and para-chain *L*_*l*_ as:

$$\begin{array}{ccc} ZC_{1}\left(M_{l}\right)=84l-60, & ZC_{1}\left(O_{l}^{1}\right)=78l-48, & \\ ZC_{1}\left(O_{l}^{2}\right)=76l-44, & ZC_{1}\left(L_{l}\right)=76l-44. & \end{array}$$

Corollary 2.2. *For a random cyclooctatetraene chain* *COC*_*l*_(*l* ≥ 3)*, the para-chain* *L*_*l*_ *and ortho chain* $O_{l}^{1}$ *, and the meta-chain* *M*_*l*_ *achieves the minimum and the maximum of* *E*(*ZC*_1_(*COC*_*l*_))*, respectively.*

Proof. Using Theorem 2.1, we acquire

$$E_{l}^{i}=E\left(ZC_{1}\left(COC_{l}\right)\right)=\left(8l-16\right)k_{1}+\left(2l-4\right)k_{2}+76l-44.$$

By taking partial derivatives, we acquire $\frac{\partial E}{\partial k_{1}}=8l-16>0$ , $\frac{\partial E}{\partial k_{2}}=2l-4>0$ . When *k*_1_ = *k*_2_ = *k*_3_ = 0 (i.e. *k*_4_ = 1), the para-chain *L*_*l*_ and ortho chain $O_{l}^{1}$ achieve the minimum of *E*(*ZC*_1_(*COC*_*l*_)), that is *COC*_*l*_ *≅L*_*l*_ or $COC_{l}≅O_{l}^{1}$ . If *k*_3_ = 1 − *k*_1_−*k*_2_ (0 ≤ *k*_1_, *k*_2_ ≤ 1), we have

$$\begin{array}{ll} E_{l}^{i} & =E\left(ZC_{1}\left(COC_{l}\right)\right)\\ & =\left(8l-16\right)k_{1}+\left(2l-4\right)k_{2}+76l-44. \end{array}$$

But *k*_1_ = *k*_2_ = 0 (when *k*_3_ = 1), *E*(*ZC*_1_(*COC*_*l*_)) can not attain the maximum value. If *k*_1_ = 1 − *k*_2_ (0 ≤ *k*_2_ ≤ 1), we acquire

$$\begin{array}{ll} E_{l}^{i} & =E\left(ZC_{1}\left(COC_{l}\right)\right)\\ & =\left(8l-16\right)\left(1-k_{2}\right)+\left(2l-4\right)k_{2}+76l-44. \end{array}$$

Therefore $\frac{\partial E}{\partial k_{2}}=-6l+12<0$ . Thus *E*(*ZC*_1_(*COC*_*l*_)) achieves the maximum value, if *k*_2_ = 0(*k*_1_ = 1), that is *COC*_*l*_ *≅M*_*l*_.

## 3 The first Zagreb connection index of a random polyphenyls chain

**FIGURE 3:**
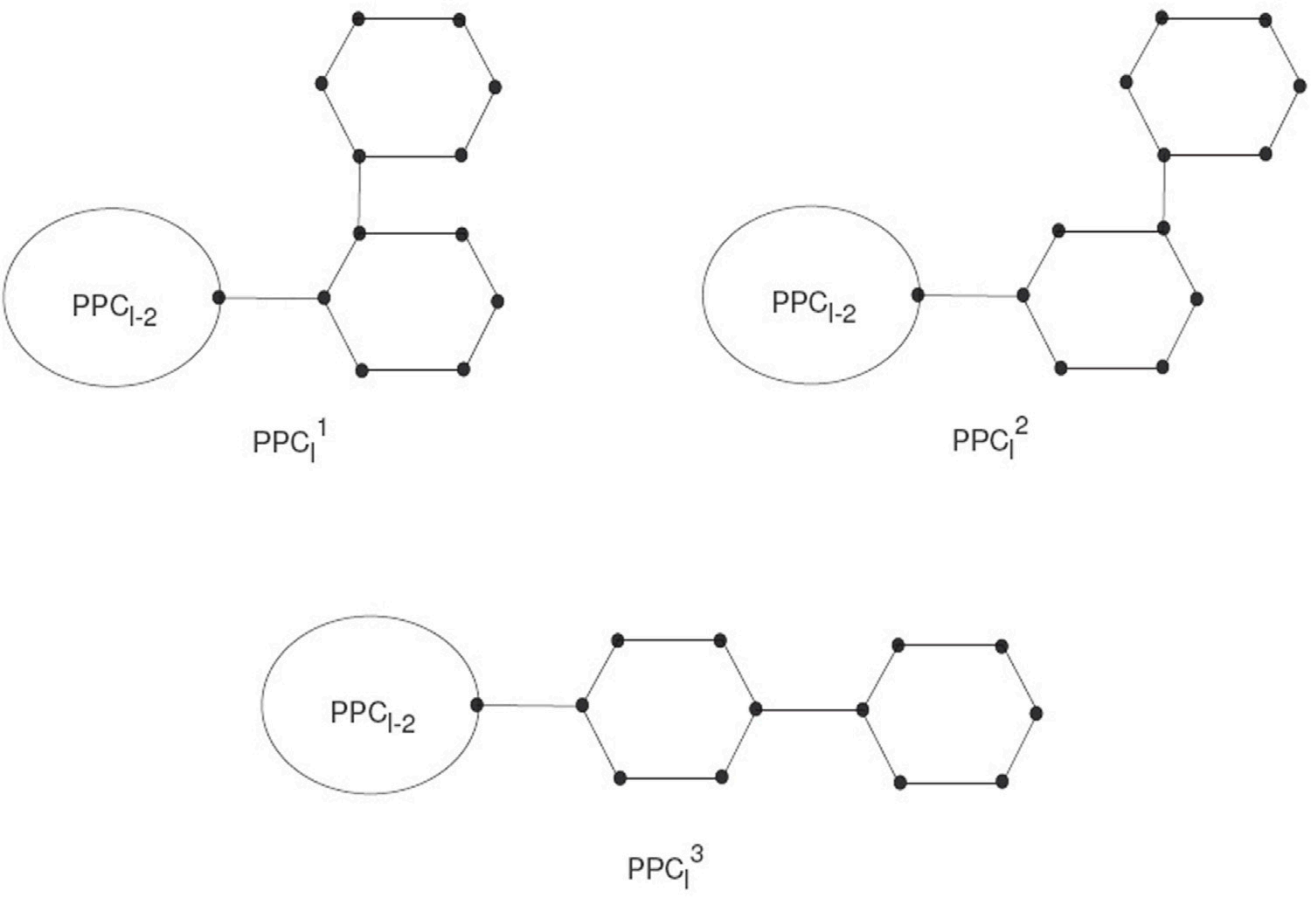
The three types of local arrangements of hexagons $PPC_{l}^{1}$ , $PPC_{l}^{2}$ , and $PPC_{l}^{3}$ .

Polyphenyls showed a molecular graph corresponding to a type of macrocyclic aromatic hydrocarbons, and these molecular graphs of polyphenyls construct a polyphenyl structure. Polyphenyls and their derivatives have applications in drug synthesis, organic synthesis, heat exchangers, *etc.,* and have received attention from chemists. A random polyphenyl chain *PPC*_*l*_ with *l* hexagons can be constructed by a polyphenyl chain *PPC*_*l*−1_ using *l*−1 hexagons attached to a new hexagon *G*_*l*_ by a bridge (see Figure 3).

**FIGURE 4:**
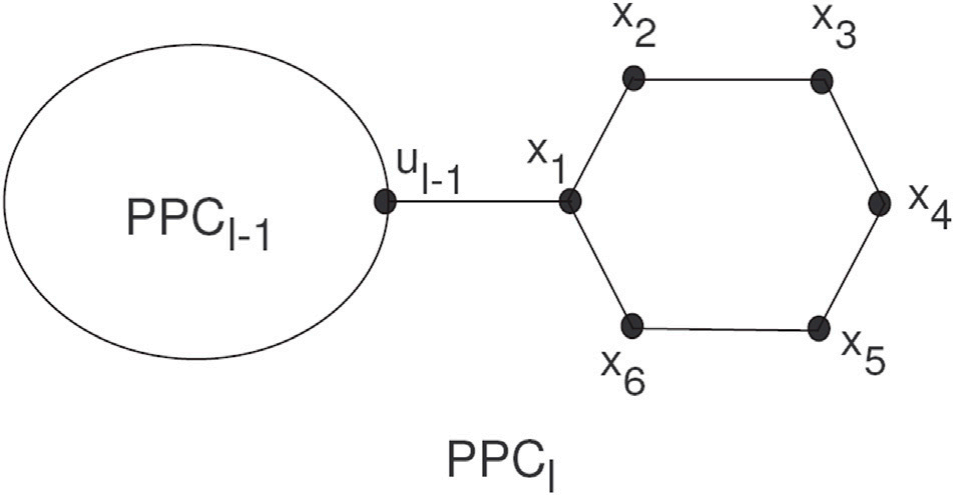
A random polyphenyl chain *PPC*_*l*_.

The *PCC*_*l*_ will be a polyphenyl chain with *l* ≥ 2 having *G*_1_, *G*_2_, *…* , *G*_*l*_ hexagons. *PPC*_*l*_ is the meta-chain *M*_*l*_, the ortho-chain $O_{l}^{1}$ and the para-chain *L*_*l*_. The new hexagon can be joined in three arrangements, which construct the local orderings. We use these as $PPC_{l}^{1}$ , $PPC_{l}^{2}$ , $PPC_{l}^{3}$ (see Figure 4).

A random polyphenyl chain *PPC*_*l*_(*k*_1_, *k*_2_) is a polyphenyl chain constructed by step-by-step attachment of new hexagons. At every step *p* = 2, 3, *…* , *l*, a random choice construct one of the three possible chains:

1 $PPC_{p-1}\to PPC_{p}^{1}$ with probability *k*_1_,
2 $PPC_{p-1}\to PPC_{p}^{2}$ with probability *k*_2_,
3 $PPC_{p-1}\to PPC_{p}^{3}$ with probability *k*_3_ = 1 − *k*_1_−*k*_2_,

Where all the given probabilities are constant. In this section, we discuss the expected value for the first Zagreb connection index of the random polyphenyl chain with *l* hexagons.

Theorem 3.1 *For* *l* ≥ 2*, the expected value for the first Zagreb connection index of the random polyphenyl chain* *PPC*_*l*_ *is*

$$E\left(ZC_{1}\left(PPC_{l}\right)\right)=\left(8k_{1}+2k_{2}+68\right)l-16k_{1}-4k_{2}-44.$$

Proof. **Case-I:** When *l* = 2, one can get

$$E\left(ZC_{1}\left(PPC_{l}\right)\right)=6\times {\left(2\right)}^{2}+4\times {\left(3\right)}^{2}+2\times {\left(4\right)}^{2}=92.$$

**Case-II:** When *l* ≥ 3, it is obvious that *f*_2_(*PPC*_*l*_), *f*_3_(*PPC*_*l*_), *f*_4_(*PPC*_*l*_) and *f*_5_(*PPC*_*l*_) depends on the four possible cases, as follows:

1 If $PPC_{l-1}\to PPC_{l}^{1}$ having probability *k*_1_, we acquire

f2PPCl1=f2PPCl−1+2,f3PPCl1=f3(l−1)+2,f4PPCl1=f4PPCl−1=2,f5PPCl1=f5PPCl−1+2.

By using the above values in Eq. 1.1, we get

$$\begin{array}{ll} ZC_{1}\left(PPC_{l}^{1}\right) & =ZC_{1}\left(PPC_{l-1}\right)+2\times 2^{2}+2\times 3^{2}\\ & \;+2\times 5^{2}\\ & =ZC_{1}\left(PPC_{l-1}\right)+76. \end{array}$$

2 If $PPC_{l-1}\to PPC_{l}^{2}$ having probability *k*_2_, we acquire

$$\begin{array}{ll} f_{2}\left(PPC_{l}^{2}\right) & =f_{2}\left(PPC_{l-1}\right)+1,\\ f_{3}\left(PPC_{l}^{2}\right) & =f_{3}\left(PPC_{l-1}\right)+2,\\ f_{4}\left(PPC_{l}^{2}\right) & =f_{4}\left(PPC_{l-1}\right)+3, \end{array}$$

By using the above values in Eq. 1.1, we get

$$\begin{array}{ll} ZC_{1}\left(PPC_{l}^{2}\right) & =ZC_{1}\left(PPC_{l-1}\right)+1\times 2^{2}+2\times 3^{2}\\ & \;+3\times 4^{2}\\ & =ZC_{1}\left(PPC_{l-1}\right)+70. \end{array}$$

3 If $PPC_{l-1}\to PPC_{l}^{3}$ having probability *k*_3_, we acquire

$$\begin{array}{ll} f_{2}\left(PPC_{l}^{3}\right) & =f_{2}\left(PPC_{l-1}\right)=6,\\ f_{3}\left(PPC_{l}^{3}\right) & =f_{3}\left(PPC_{l-1}\right)+4,\\ f_{4}\left(PPC_{l}^{3}\right) & =f_{4}\left(PPC_{l-1}\right)+2, \end{array}$$

By using the above values in Eq. 1.1, we get

$$\begin{array}{ll} ZC_{1}\left(PPC_{l}^{3}\right) & =ZC_{1}\left(PPC_{l-1}\right)+4\times 3^{2}+2\times 4^{2}\\ & =ZC_{1}\left(PPC_{l-1}\right)+68. \end{array}$$

Now

$$\begin{array}{ll} E_{l}^{i} & =E\left(ZC_{1}\left(PPC_{l}\right)\right)\\ & =k_{1}ZC_{1}\left(PPC_{l}^{1}\right)+k_{2}ZC_{1}\left(PPC_{l}^{2}\right)\\ & \;+\left(1-k_{1}-k_{2}\right)ZC_{1}\left(PPC_{l}^{3}\right)\\ & =ZC_{1}\left(PPC_{l-1}\right)+8k_{1}+2k_{2}+68. \end{array}$$ (3.1)

Note that $E\left[E_{l}^{i}\right]=E_{l}^{i}$ . By applying the expression operator to Eq. 3.1 and also *l* ≥ 3, we get

$$E_{l}^{i}=E_{l-1}^{i}+8k_{1}+2k_{2}+68.$$ (3.2)

The result Eq. 3.2 is a first-order non-homogeneous linear difference equation with constant coefficients. The general solution of the homogeneous side is Eq. 3.2 is *E* ^*i*^ = *C*. Suppose *E* ^*i*^′ = *bl* is a particular result of Eq. 3.2, using *E* ^*i*^′ into Eq. 3.2, we acquire

$$b=8k_{1}+2k_{2}+68.$$

Finally the general solution of Eq. 3.2 is given by

$$\begin{array}{ll} E_{l}^{i} & =E^{i}+E^{i^{\prime }}\\ & =E\left(ZC_{1}\left(PPC_{l}\right)\right)=\left(8k_{1}+2k_{2}+68\right)l+C. \end{array}$$

Applying the initial condition *l* = 3, we get following

$$C=-16k_{1}-4k_{2}-44.$$

Therefore

$$E_{l}^{i}=E\left(ZC_{1}\left(PPC_{l}\right)\right)=\left(8k_{1}+2k_{2}+68\right)l-16k_{1}-4k_{2}-44.$$

If *k*_1_ = 1 (respectively, *k*_2_ = 1) and *k*_2_ = *k*_3_ = 0 (respectively, *k*_1_ = *k*_3_ = 0), then *PPC*_*l*_ = *M*_*l*_ (respectively, *PPC*_*l*_ = *O*_*l*_). Similarly, if *k*_3_ = 1 and *k*_1_ = *k*_2_ = 0, then *PPC*_*l*_ = *L*_*l*_. By Theorem 3.1, we can acquire the first Zagreb connection index of polyphenyl chains like meta *M*_*l*_, ortho *O*_*l*_, and para *L*_*l*_, as

$$\begin{array}{ll} & ZC_{1}\left(M_{l}\right)=76l-60,\;ZC_{1}\left(O_{l}\right)=70l-48,\\ & ZC_{1}\left(L_{l}\right)=68l-44. \end{array}$$

Corollary 3.2. F*or a random polyphenyl chain* *PPC*_*l*_(*l* ≥ 3)*, the para-chain* *L*_*l*_ *and the meta-chain* *M*_*l*_ *achieves the minimum and the maximum* *E*(*ZC*_1_(*PPC*_*l*_))*, respectively.*

Proof. From Theorem 3.1, we obtain

$$\begin{array}{ll} E_{l}^{i} & =E\left(ZC_{1}\left(PPC_{l}\right)\right)\\ & =\left(8l-16\right)k_{1}+\left(2l-4\right)k_{2}+68l-44. \end{array}$$

By taking partial derivatives, we acquire $\frac{\partial E}{\partial k_{1}}=8l-16>0$ , $\frac{\partial E}{\partial k_{2}}=2l-4>0$ . When *k*_1_ = *k*_2_ = 0 (i.e. *k*_3_ = 1), the para-chain *L*_*l*_ has the minimum of *E*(*ZC*_1_(*COC*_*l*_)), that is *PPC*_*l*_ *≅L*_*l*_. If *k*_1_ = 1 − *k*_2_ (0 ≤ *k*_2_ ≤ 1), we acquire

$$\begin{array}{ll} E_{l}^{i} & =E\left(ZC_{1}\left(PPC_{l}\right)\right)\\ & =\left(8l-16\right)\left(1-k_{2}\right)+\left(2l-4\right)k_{2}+68l-44. \end{array}$$

Therefore $\frac{\partial E}{\partial k_{2}}=-6l+12<0$ . Thus *E*(*ZC*_1_(*PPC*_*l*_)) achieves the maximum value, if *k*_2_ = 0(*k*_1_ = 1), that is *PPC*_*l*_ *≅M*_*l*_.

## 4 The first Zagreb connection index of random chain network *PG*_*l*_

**FIGURE 5:**
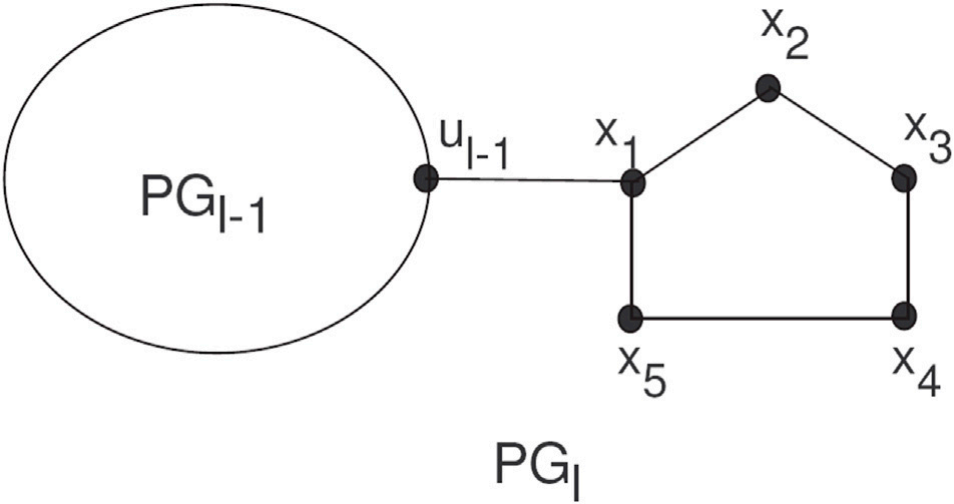
A random chain networks *PG*_*l*_.

The random chain networks *PG*_*l*_ with *l* pentagons can be constructed by *PG*_*l*−1_ having *l*−1 pentagons attached to a new pentagon *H*_*l*_ by a bridge (see Figure 5).

**FIGURE 6:**
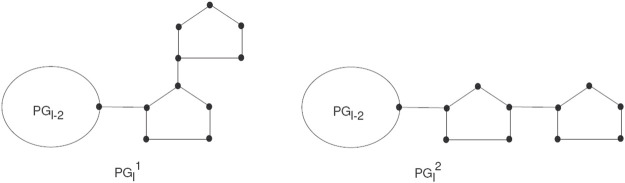
The two types of local arrangements of pentagons $PG_{l}^{1}$ and $PG_{l}^{2}$ .

The *PG*_*l*_ will be a random chain network with *l* ≥ 2, and *H*_1_, *H*_2_, *…* , *H*_*l*_ pentagons. For *l* ≥ 3, there are two ways to attach pentagons at the end and get $PG_{l}^{1}$ and $PG_{l}^{2}$ , (see Figure 6). For such a random chain network, any step for *q* = 2, 3, 4, *…* , *l* can be constructed by two possible chains with given probabilities *k*_1_ and *k*_2_, respectively:

1 $PG_{q-1}\to PG_{q}^{1}$ with probability *k*_1_,
2 $PG_{q-1}\to PG_{q}^{1}$ with probability *k*_2_ = 1 − *k*_1_,

Where all the given probabilities are constant.

This section discusses the expected value for the first Zagreb connection index of the random chain network with *l* pentagons. The proof of Theorem 4.1 is the same as the proofs of Theorem 2.1 and Theorem 3.1; therefore, we omit it here.

Theorem 4.1 *For* *l* ≥ 2*, the expected value for the first Zagreb connection index of random chain network* *PG*_*l*_ *is* *E*(*ZC*_1_(*PG*_*l*_)) = (6*k*_1_+66)*L*−12*k*_1_−48*.*

If *k*_1_ = 1 (respectively, *k*_2_ = 1) and *k*_2_ = 0 (respectively, *k*_1_ = 0), then $PG_{l}=PG_{l}^{1}$ (respectively, $PG_{l}=PG_{l}^{2}$ ). By Theorem 4.1, we can acquire the first Zagreb connection index of the meta-chain $PG_{l}^{1}$ and para-chain $PG_{l}^{2}$ , as

$$ZC_{1}\left(PG_{l}^{1}\right)=72l-60,\;ZC_{1}\left(PG_{l}^{2}\right)=66l-48.$$

Corollary 4.2. *For a random chain network* *PG*_*l*_(*l* ≥ 3)*, the para-chain* $PG_{l}^{2}$ *and the meta-chain* $PG_{l}^{1}$ *achieves the minimum and the maximum of* *E*(*ZC*_1_(*PG*_*l*_))*, respectively.*

## 5 The average values for the first Zagreb connection index

This section finds the average values for the first Zagreb connection index concerning the sets of all cyclooctatetraene chains with *l* octagons, polyphenyl chains with *l* hexagons, and chain networks with *l* pentagons. Let $G_{l}$ , $R_{l}$ and $Q_{l}$ be the sets of all cyclooctatetraene chains, polyphenyl chains, and random chain network, respectively. The average values for the first Zagreb connection index for the sets $G_{l}$ , $R_{l}$ and $Q_{l}$ are given below:

$$\begin{array}{ll} & ZC_{1}^{\mathrm{avg}}\left(G_{l}\right)=\frac{1}{G_{l}}\underset{H\in G_{l}}{\sum }ZC_{1}\left(H\right),\\ & ZC_{1}^{\mathrm{avg}}\left(R_{l}\right)=\frac{1}{R_{l}}\underset{H\in R_{l}}{\sum }ZC_{1}\left(H\right),\\ & ZC_{1}^{\mathrm{avg}}\left(Q_{l}\right)=\frac{1}{Q_{l}}\underset{H\in Q_{l}}{\sum }ZC_{1}\left(H\right). \end{array}$$

The average value concerning sets $G_{l}$ , $R_{l}$ , and $Q_{l}$ are expected values for the first Zagreb connection index of the random chains. From Theorem 2.1, Theorem 3.1 and Theorem 4.1, we have.

Theorem 5.1 *The average value for the first Zagreb connection index concerning the set* $G_{l}$ *is given as:*

$$ZC_{1}^{\mathrm{avg}}\left(G_{l}\right)=\frac{157}{2}l-49.$$

After calculation, we acquire

$$\begin{array}{ll} ZC_{1}^{\mathrm{avg}}\left(G_{l}\right) & =\frac{1}{4}\left(ZC_{1}\left(M_{l}\right)+ZC_{1}\left(O_{l}^{1}\right)\right.\\ & \left.\;+ZC_{1}\left(O_{l}^{2}\right)+ZC_{1}\left(L_{l}\right)\right). \end{array}$$

Theorem 5.2 T*he average value for the first Zagreb connection index concerning* $R_{l}$ *is*

$$ZC_{1}^{\mathrm{avg}}\left(R_{l}\right)=\frac{214}{3}l-\frac{152}{3}.$$

After calculation, we acquire

$$\begin{array}{ll} ZC_{1}^{\mathrm{avg}}\left(R_{l}\right) & =\frac{1}{3}\left(ZC_{1}\left(M_{l}\right)+ZC_{1}\left(O_{l}\right)\right.\\ & \left.\;+ZC_{1}\left(L_{l}\right)\right). \end{array}$$

Theorem 5.3 *The average value for the first Zagreb connection index concerning* $Q_{l}$ *is* $ZC_{1}^{\mathrm{avg}}\left(Q_{l}\right)=69l-54$ *. It is also:*

$$ZC_{1}^{\mathrm{avg}}\left(Q_{l}\right)=\frac{1}{2}\left(ZC_{1}\left(PG_{l}^{1}\right)+ZC_{1}\left(PG_{l}^{2}\right)\right).$$

## 6 Conclusion

This study computed the expected values of the first Zagreb connection index in a random cyclooctatetraene chain, random polyphenyls chain, and random chain network with *l*, octagons, hexagons, and pentagons, respectively. It has discussed the maximum chain and the minimum chain of the *COC*_*l*_, *PPC*_*l*_, and *PG*_*l*_, respectively, concerning the expected values of these chains. The average values discussed in all of the above are considered random chains for unique chains.

## Data Availability

The original contributions presented in the study are included in the article/Supplementary Material, further inquiries can be directed to the corresponding authors.
